# Long-term trends in evolution of indels in protein sequences

**DOI:** 10.1186/1471-2148-7-19

**Published:** 2007-02-13

**Authors:** Yuri Wolf, Thomas Madej, Vladimir Babenko, Benjamin Shoemaker, Anna R Panchenko

**Affiliations:** 1 National Center for Biotechnology Information, National Institutes of Health, Bethesda, 20894, US; 2 Institute of Cytology and Genetics, Novosibirsk, Russia

## Abstract

**Background:**

In this paper we describe an analysis of the size evolution of both protein domains and their indels, as inferred by changing sizes of whole domains or individual unaligned regions or "spacers". We studied relatively early evolutionary events and focused on protein domains which are conserved among various taxonomy groups.

**Results:**

We found that more than one third of all domains have a statistically significant tendency to increase/decrease in size in evolution as judged from the overall domain size distribution as well as from the size distribution of individual spacers. Moreover, the fraction of domains and individual spacers increasing in size is almost twofold larger than the fraction decreasing in size.

**Conclusion:**

We showed that the tolerance to insertion and deletion events depends on the domain's taxonomy span. Eukaryotic domains are depleted in insertions compared to the overall test set, namely, the number of spacers increasing in size is about the same as the number of spacers decreasing in size. On the other hand, ancient domain families show some bias towards insertions or spacers which grow in size in evolution. Domains from several Gene Ontology categories also demonstrate certain tendencies for insertion or deletion events as inferred from the analysis of spacer sizes.

## Background

Proteins evolve through gene duplication, diversification and domain shuffling, which allow novel proteins to emerge via different domain combinations. Many evolutionary mechanisms shaping protein sequence and structure can be probed by studying the length distributions of proteins and protein domains. Fusion of single domain proteins and domain accretion, for example, play an important role in the evolution of eukaryotic proteins [1-3]; as a result eukaryotic proteins on average are longer than bacterial and archaeal proteins [4-6]. Moreover, it was shown previously that there exists a correlation between sequence length and protein conservation [7,8], sequence length and protein expression [9].

Diverse multi-domain proteins may consist of homologous domains and knowledge of protein domain evolution may considerably help in reconstructing the evolutionary history of entire proteins. Changes in protein domains result mostly from point mutations, insertion and deletion processes. Although amino acid insertion and deletion (indel) events in proteins are less frequent than amino acid substitutions [10-12], they can have a major effect in metazoan protein evolution and indel bias can influence the overall genome size [13-15]. It has been observed that indels most often occur in non-conserved protein loop regions. While proteins seem to be rather tolerant to indels in loops compared to core structure elements, protein loops can not be viewed as "random coils" and indels are under constant evolutionary pressure [16]. At the same time, indels can relax structural tension occurring due to amino acid substitutions in some proteins and can lead to significant structural changes [17].

The mechanisms of indel events are not very well understood and there are only few statistical models describing these events in evolution [18-20]. Traditionally, indels in sequence alignments are scored using affine gap penalties despite the fact that this model does not adequately describe the evolution of insertions and deletions. In particular, the empirical distribution of indel lengths was analyzed for the alignment of closely related proteins and it was shown that it can be well approximated by the Zipfian distribution [10,21,22]. It has also been found that the probability of a gap in the alignment of two homologous sequences depends on the evolutionary distance and there exists a strong relationship between the evolutionary distance and the indel lengths [10,11].

In this paper we analyze the size evolution of whole protein domains and indels in protein domains, as judged by changing sizes of whole domains or individual unaligned regions. We study intra-domain events which are not affected by domain shuffling and domain accretion in multidomain proteins. To examine these events on a wide scale of evolutionary distances we use the Conserved Domain Database (CDD) [23] that provides accurate domain alignments of diverse sequences. We are interested in whether the insertion and deletion events in protein domains as a whole or in individual fragments are balanced and if there exist trends toward increasing or decreasing indel or domain lengths. To answer these questions, we perform an extensive analysis of protein domain families spanning a wide range of different taxonomic and functional categories. The answers which we provide in this paper give the means to model indel events in the evolution of different domain families and to understand the nature of protein domain size diversity.

## Results

### Domain size evolution

**Figure 1:**
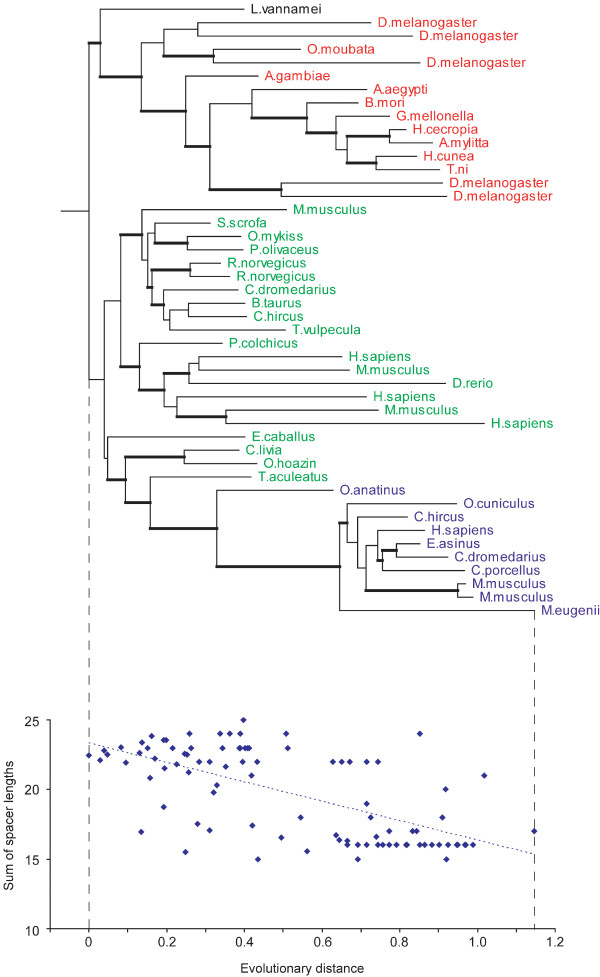


**Figure 2:**
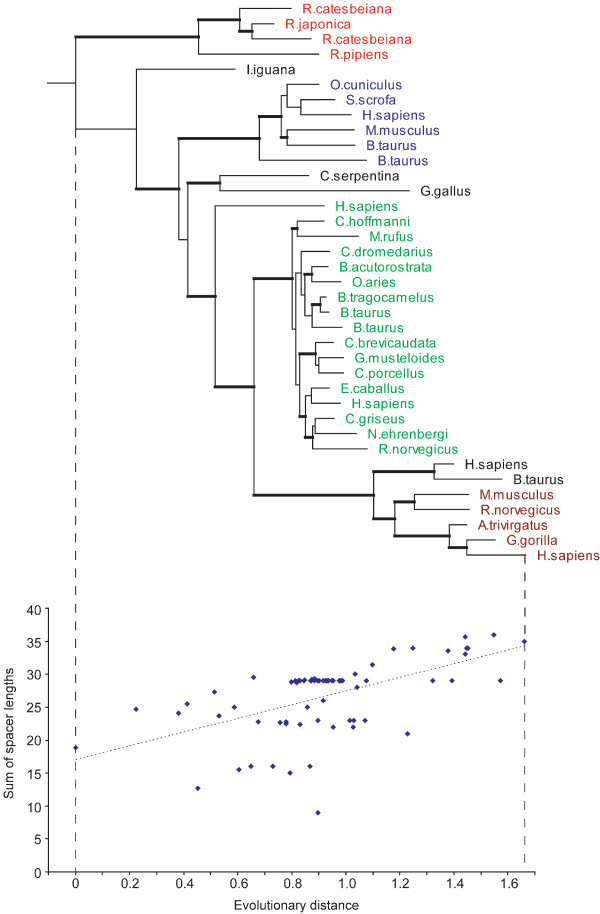


Protein domain size evolution was studied by examining the correlation between the domain size (which is inferred from the sum of the spacer lengths; block elements give a constant contribution to the size for all sequences from the same CD family) and the evolutionary distance from the root of the tree to a given node. We note that there are other domain classifications [24,25] which may define domain boundaries somewhat differently, but none of them provides conserved domain definitions with the block-based multiple sequence alignments necessary for our study. The regression coefficients (slopes) were calculated by approximating this dependence with the linear model and by quantifying the rate of domain size change in evolution in terms of the number of residues per unit of evolutionary distance. The sign of its regression coefficient indicates the tendency of the domain to increase/decrease in size. As a result of the correlation analysis we found that 59–63% (the range is given for two internal node mapping models) of all domains do not show a statistically significant correlation while for 37–41% of the domains the size does correlate with evolutionary distance (with P-values < 0.01). According to the stringent multiple comparison Bonferroni test and modified Bonferroni tests [26] the fraction of domains with statistically significant correlations is about 19–25%. Among domain families exhibiting particularly good linear correlations were tubulin/Ftsz (cd00286, ρ = 0.94), Phosphoribosyltransferase (cd00516, ρ = 0.77), HMG-CoA_reductase (cd00365, ρ = -0.83) and LMWPc (cd00115, ρ = -0.70). Two examples of positive and negative correlations are shown in Figures 1 and 2.

The first example represents the Ribonuclease A family (cd00163, Figure 1), which shows a significant trend to increase domain sizes (inferred from the total sizes of all spacers in the domain alignment) (ρ = 0.57). As can be seen from this Figure, the frog ribonucleases (red) have the smallest domains with about 9–16 residues in spacer regions, angiogenins (blue, 22–23 residues) and the mammalian RNase 1 (green, 28–30 residues) have intermediate size domains and finally the eosinophil ribonucleases (brown) have the largest domains with about 33–36 residues in spacer regions. The second example of the Lysozyme/Lactalbumin domain family (cd00119, Figure 2) shows a negative correlation with respect to domain sizes. In this family there are three main groups, the mammalian lysozymes (green), the insect lysozymes (red), and the mammalian alpha-lactalbumins (blue). The mammalian lysozymes are nearest to the root of the tree and have the largest domains. The insect lysozymes and alpha-lactalbumins are at about the same distance from the root and they have similar domain sizes, smaller than the mammalian lysozymes.

**Figure 3:**
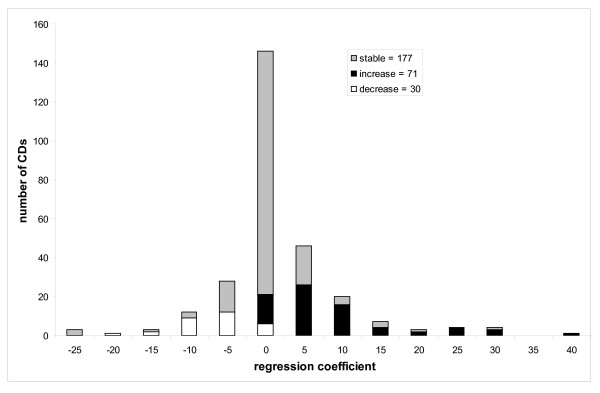


Figure 3 shows the histogram of regression coefficients using model #2 for domains which increase (black shading), decrease (in white) in size and those that do not change in size (so called "stable" domains with no statistically significant tendency to change the domain size, grey shading). As can be seen from this figure, the fraction of domains having a significant tendency to increase in size (25–29%) is twofold larger than the fraction of domains decreasing in size (11–12%). A modified Bonferroni test yields 14–18% domains increasing in size and 5–8% domains decreasing in size respectively. Limiting the correlation analysis only to the internal nodes results in 25–31% of domains increasing in size versus 12–19% of domains decreasing in size.

Assuming equal distribution of families among these two increasing and decreasing classes we expect that, half of families (51 out of 102 for model #2) will exhibit an increasing pattern and another half will decrease in size. However, we observed 71 cases instead of 51 and the probability to observe such bias given the above assumption can be estimated from the binomial distribution and is very small (P < 0.00005). We also estimated an average rate of domain size change which was found to be 7.2 (5.7) residues per domain per unit of evolutionary distance for domains of increasing and decreasing size respectively. Unlike for the number of domains, there is no statistically significant difference between the two average rates.

Analyzing domain families with large spacers of more than 50 residues long (so-called "inserted domains", 32 domains altogether), we investigated the functional and taxonomic assignments for these domains and found that 27 of them represent enzyme domains and 31 of them belong to the "Root" taxonomic category (with the background of 117 enzyme domain families and 183 ancient domain families in the overall test set). The probability to observe such bias, given the assumption that families with "inserted domains" are distributed equally among different functional/taxonomic classes, can be estimated from the binomial distribution, which yields a p-value of < 10^-6 ^for enzymatic domains and a p-value < 3*10^-5 ^for ancient domains. Thus, ubiquitous domains with enzymatic activity have a tendency to accommodate very long indels, a similar observation for enzymes having been reported earlier [27]. In our dataset we found that the long insertions predominate over long deletions, the number of enzymatic domains with spacers longer than 50 residues inserted (12 domains) is three times larger than the number of domains with long spacers deleted in evolution (4 domains). In general, the evolutionary mechanism of inserting the whole domain into another protein domain might be different from the mechanisms of short indel evolution, but excluding those domain families with spacers longer than 50 residues does not change the overall conclusions reported in this paper (data not shown).

### Spacer size evolution

**Figure 4:**
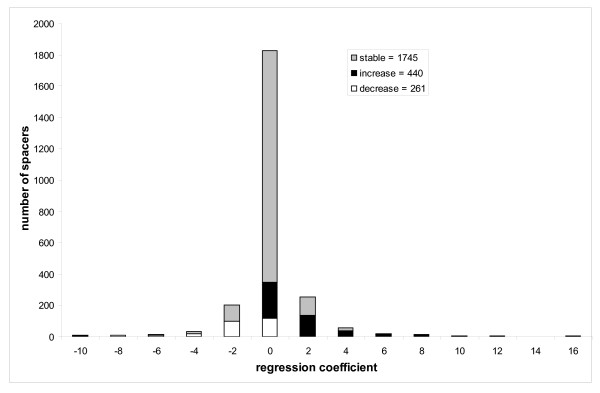


We assigned individual spacers to the functional and taxonomic categories of corresponding CDs and performed an analysis similar to that described in the previous section with the only difference that instead of domain sizes, the individual spacer lengths were mapped to the nodes of phylogenetic trees. Using this mapping we can calculate the regression coefficients for each individual spacer in each domain. Figure 4 shows the histogram of regression coefficients for individual spacers from the entire test set (2451 individual spacers), among them 67–71% (depending on the internal node mapping model) are "stable" and do not exhibit any statistically significant tendency to increase/decrease in size with evolutionary distance while 29–33% of spacers show significant correlation (P < 0.01). Similar to the trend observed for entire domain sizes, the majority of spacers (18–21%) systematically increase their size in the course of evolution while a small fraction of them (11–12%) decrease in size (the probability to observe such bias is < 7*10^-11^). Although the number of spacers which increase in size exceeds the number of spacers which decrease in size, the rate of spacer size change is approximately equal, 1.8(1.9) amino acids per spacer per unit of evolutionary distance.

**Figure 5:**
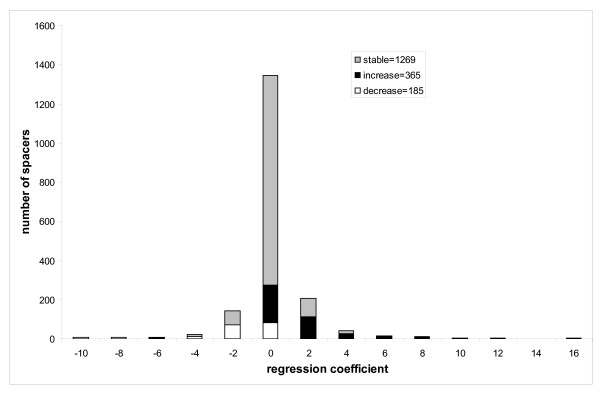


**Figure 6:**
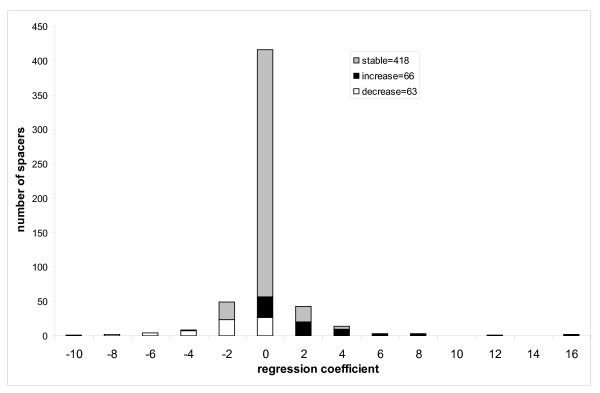


**Figure 7:**
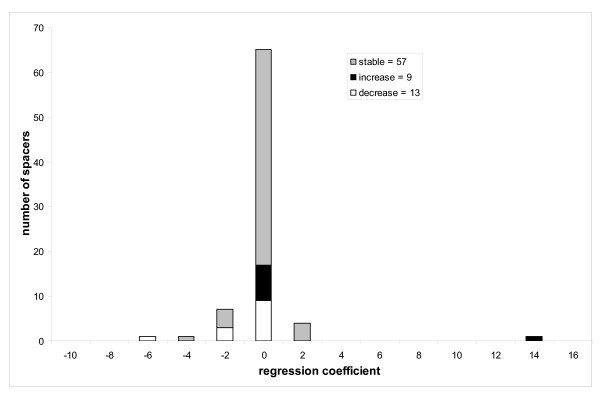


To analyze whether the tendency of spacers to increase/decrease in size depends on the taxonomic or functional category we performed the Pearson chi-squared test. First we checked the null hypothesis that the assignment to taxonomy groups ("E", "B" and "R", see Methods) is not correlated with the sign of the regression coefficients. We showed that the null hypothesis is rejected with p-value < 0.0003, which indicates that different taxonomic groups overall have different tendencies in spacer size evolution. For example, the spacers from ancient domain families show some bias toward the spacers which grow in size (contributing 19% of the overall value of χ^2 ^statistics, Figure 5). On the other hand, eukaryotes are depleted in spacers which increase in size compared to the overall test set (the number of spacers with statistically significant positive correlation is almost equal to the number of spacers with negative correlation, Figure 6). In particular, the contribution to the χ^2 ^value attributed to the eukaryotic spacers accounts for 46% of the overall χ^2 ^value of the test. For bacterial families (which are underrepresented in our test set), the situation is reversed and the majority of spacers decrease in size (although the bacterial spacers contribute only 11% of the overall χ^2 ^value, Figure 7).

Different protein domains can have different tolerances to insertion or deletion events depending on domain function and localization in a cell. We investigated this by annotating protein domains with respect to the Gene Ontology (GO) classification [28] and tested the null hypothesis that the sample of a given GO term represents an unbiased sample from the overall test set of individual spacers. We calculated the χ^2^-value for those GO categories with more than 8 expected values and estimated p-values under the null hypothesis; Table 1 lists those GO categories with a p-value of less than 0.01 and those categories which contribute more than 2/3 of the χ^2 ^value due to excess (red) or shortage (blue) of observed families are highlighted. As can be seen from this table, proteins with functions related to transferase activity, replicative cell aging, positive regulation of cell proliferation and transcription all have a statistically significant excess of spacers increasing in size. On the other hand, domains from proteins located in the endoplasmic reticulum membrane have an excess of spacers decreasing in size. Proteins participating in chemotaxis, protein folding and G-protein coupled signaling pathways have mostly "stable" domains while proteins from the ubiquitin cycle and ubiquitin dependent catabolism, membrane fraction, mitosis and G-protein coupled signaling pathways are deprived of spacers decreasing in size. We also investigated whether spacer size increase/decrease can be attributed to the number of interactions with other proteins/domains. The analysis performed using conserved binding modes [29] did not find any statistically significant correlation between the trend of the size change and the number of interacting partners for a given domain or spacer region.

**Table 1:** GO categories listed for domain families with statistically significant bias (p-value < 0.01) with respect to increasing, decreasing and stable individual spacers.

**Decreasing**	**Stable**	**Increasing**	**GO annotation**	**P-value**
6 (9.8)	47 (64.9)	**38 (16.3)**	transferase activity, transferring glycosyl groups	0.0000
12 (11.2)	52 (74.2)	**40 (18.7)**	replicative cell aging	0.0000
**25 (11.7)**	58 (77.7)	26 (19.6)	endoplasmic reticulum membrane	0.0000
24 (20.6)	112 (136.9)	**56 (34.5)**	positive regulation of cell proliferation	0.0001
*0 (11.7)*	93 (77.7)	16 (19.6)	ubiquitin cycle	0.0005
*14 (32.1)*	231 (213.2)	54 (53.7)	membrane fraction	0.0029
5 (8.6)	70 (57.1)	5 (14.4)	chemotaxis	0.0052
*0 (8.9)*	66 (59.2)	17 (14.9)	ubiquitin-dependent protein catabolism	0.0068
*4 (13.5)*	91 (89.9)	31 (22.6)	mitosis	0.0074
30 (33.5)	205 (222.5)	**77 (56)**	transcription	0.0082
2 (8.2)	66 (54.2)	8 (13.6)	protein folding	0.0084
*4 (14.9)*	111 (99.1)	24 (25)	G-protein coupled receptor protein signaling pathway	0.0089

First three columns show the observed and expected number (in parentheses) of decreasing, stable and increasing spacers for different GO categories. Categories, contributing more than 2/3 of the χ^2 ^value due to excess (in bold) or shortage (in italic) of observed families are highlighted. The last column lists p-value of Pearson χ^2^-test.

## Discussion

The collection of accurate, curated multiple sequence alignments from CDD gives us an opportunity to study the evolution of domain and spacer sizes on a wide scale of evolutionary distances. Thus far the indel events have been studied on sets of relatively closely related species such as human, mouse and rat, where indels can be defined explicitly. It has been found that there exists a 2–3 fold excess of deletions over insertions in non-coding regions (and pseudogenes in particular) from human and murids [15,30-32] and much higher deletion bias in *Drosophila melanogaster *pseudogenes [33]. Moreover, deletions are approximately three times more common than insertions in loci causing Mendelian diseases [12]. Protein coding regions, however, are generally under higher selective pressure than pseudogenes and non-coding regions and as was shown in a recent studies the ratio of deletions to insertions in protein coding regions is much closer to unity compared to non-coding regions. For example, a deletion to insertion ratio of microindels (upto 10–15 bp) in non-coding regions of mouse is 2.5 : 1 and this ratio is reduced to a 1.1(1.05) : 1 in protein coding regions [34,35]. Along these lines, we showed that for eukaryotic domains there exists a bias towards deletions (spacers decreasing in size) compared to the overall test set, which is significantly enriched with insertions (spacers increasing in size). As a result, for eukaryotes from our test set the total number of spacers which grow in size in evolution is approximately equal to the number of spacers which decrease in size.

For the entire test set we observed a certain pattern for domains and spacers to increase in size on average, with a two-fold difference between the number of domains/spacers growing in size over those diminishing in size. The overall evolutionary scenario which we can portray based on our study is the following. It has been argued that it is unlikely that early proteins represented long peptide chains. On the contrary, various data suggest that the first protein domains emerged through the recombination of short peptides or a limited vocabulary of structural units [17,36,37]. Apparently, the spacers between the domain core structural elements were minimal in size, just enough to span the spatial gaps, connecting the structure. Then, in the course of evolution the majority of ancient domains acquired additional residues through the subsequent set of insertion events, although in a fraction of domains the equilibrium of indels tended towards deletions. A plausible explanation for the prevalence of increasing spacers and domains is the selection for acquisition of novel functions and fine-tuning of existing ones. Surprisingly, as we show, the rates of net insertion and deletion size change were not significantly different between each other with the average rate of 1.8–1.9 residues per spacer per unit of evolutionary distance. Similar observations have been made about the similarity of size distributions of insertions and deletions in three mammalian genomes [15].

For relatively "modern" proteins, however, the trend of domain size evolution was rather different. Eukaryotic proteins started losing residues in spacers, indicating that in eukaryotic evolution deletions played as important a role as insertions. Eukaryotic novel proteins seem to evolve mainly through acquiring new domains and through domain shuffling which could result in longer proteins with slightly shorter individual domains. At the same time, in bacteria the deletion trend was even more pronounced (although supported by rather limited amount of data from our test set) and, indeed, it was shown earlier that deletions in *E. coli *are 8 times more frequent than insertions [38]. Such bias in bacteria towards deletions can be explained by strong selection pressure on genome size which is primarily composed of protein coding regions [8,39].

## Conclusion

There are different factors which would favor shorter or longer proteins or spacers in evolution [40-43]. Efficiency of protein translation, transcription and the folding process would probably benefit from shorter proteins [41,42]. On the other hand, certain insertions may also be advantageous and subject to positive selection. For example, lineage-specific insertions/deletions in the elastin gene have functional importance in each lineage [44] and housekeeping proteins from pathogenic organisms may contain insertions/deletions responsible for virulence properties [45]. Our study showed that one third of all protein domains have a statistically significant linear correlation between the evolutionary distance and the domain/spacer sizes and moreover, there is a certain tendency for domain/spacer sizes to increase with evolutionary distance. We do not yet have an explanation for these observations, however, future in-depth studies may provide further insights into these phenomena.

## Methods

### Benchmark construction

The analysis was performed on a set of protein families with curated alignments from the NCBI Conserved Domain Database (CDD). CDD comprises diverse non-redundant sequences and alignments are refined using three-dimensional structures and structure-structure alignments [46]. CD alignments are block-wise multiple alignments where block regions are defined as those aligned among all family members. CD alignments are constructed to ensure enough sequence diversity and taxonomy span while avoiding bias towards highly represented sequences in the database, which is important for our analysis. The redundancy is removed by using single-linkage clustering to group the domain sequences with greater than 67% sequence identity and then choosing one representative from each preferred taxonomy node within each sequence cluster (the list of preferred taxonomy nodes can be downloaded from the CDTree [47]. We start our analysis with a set of 362 manually curated parent node alignments from CDD version 2.00 [48,49]. Parent alignments correspond to the top node alignments in the hierarchy of CD families. We excluded CD families consisting of short sequence repeats (ex. SUSHI repeats) and those containing less than 10 sequences. The redundancy between protein domain families was checked using the procedure implemented in the CDART algorithm [50]; and not more than one domain family from the same domain cluster was retained in the final test set, which yielded 278 domain families. A table is available listing the 278 test domains with taxonomy assignments and computed regression coefficients [51].

The domain families from the test set encompass a large spectrum of functional and taxonomic groups. Protein function was categorized by the Gene Ontology (GO) terms [28]. Gene ontology (GO) annotations were obtained from GenBank for individual family members and pooled for the whole family. The taxonomic information for each CD family was assigned according to the range of organisms in which the family members were represented [52]. We used a simplified classification of the families into the following three categories: "R" ("Root", family members are present in at least two kingdoms among eukaryotes, prokaryotes and archaea and thus thought to be of ancient origin, dating back at least to the Last Universal Common Ancestor; 182 families); "E" (eukaryote-specific protein families; 85 families) and "B" (bacteria-specific protein families; 11 families). There were no archaea-specific families in our dataset.

Phylogenetic trees were constructed from the aligned block regions (in case of sequence repeats only one instance was kept) by the neighbor-joining method [53] with the PHYLIP package [54]. Blocks represent regions where all CDD sequences are aligned so that the resulting trees are not in any case dependent on the difference between spacer's lengths. The neighbor joining trees were rooted manually using the taxonomy of represented organisms. If multiple subfamilies within a protein domain family were present, the root was placed on the deepest inter-subfamily branch so as to balance the average length between the root and every external node of each subtree. For about 30% of the trees an alternative root placement was checked and it was observed that the overall results do not change if alternatively rooted trees were used. The phylogenetic trees are available at the ftp site [55].

### Spacer length calculation

Taking advantage of CDD block structure where multiple alignments are anchored at certain conserved positions, we define a *spacer *as a non-aligned segment between two consecutively aligned block elements. To analyze how spacer lengths change in the course of evolution, the spacer lengths for all CDD sequences between two consecutive blocks were individually mapped to the external nodes of the phylogenetic tree for the corresponding domain family. To study the evolution of domain sizes, the sum of all spacer lengths in a CDD alignment (not counting N-terminal and C-terminal spacers) was mapped to the external nodes of the phylogenetic tree.

The values of spacer lengths were inferred for the internal nodes of the phylogenetic trees using the following models. According to model #1, the spacer length for an internal node was inferred to be the same as the phylogenetically closest external node. Model #2 (analogous to the squared-change parsimony,[56], Ch. 23) inferred the spacer lengths for internal nodes as a weighted average of the spacer lengths of external nodes using a recursive procedure. For a strictly binary tree we can define:

si=s1h2+s2h1h1+h2
 MathType@MTEF@5@5@+=feaafiart1ev1aaatCvAUfKttLearuWrP9MDH5MBPbIqV92AaeXatLxBI9gBaebbnrfifHhDYfgasaacH8akY=wiFfYdH8Gipec8Eeeu0xXdbba9frFj0=OqFfea0dXdd9vqai=hGuQ8kuc9pgc9s8qqaq=dirpe0xb9q8qiLsFr0=vr0=vr0dc8meaabaqaciaacaGaaeqabaqabeGadaaakeaacqWGZbWCdaWgaaWcbaGaemyAaKgabeaakiabg2da9maalaaabaGaem4Cam3aaSbaaSqaaiabigdaXaqabaGccqWGObaAdaWgaaWcbaGaeGOmaidabeaakiabgUcaRiabdohaZnaaBaaaleaacqaIYaGmaeqaaOGaemiAaG2aaSbaaSqaaiabigdaXaqabaaakeaacqWGObaAdaWgaaWcbaGaeGymaedabeaakiabgUcaRiabdIgaOnaaBaaaleaacqaIYaGmaeqaaaaaaaa@41A8@

Where *s*_*i *_is the spacer length at internal node *i*; *s*_1 _and *s*_2 _the spacer lengths assigned to the direct descendants of node *i*; and *h*_1 _and *h*_2 _the heights of the descendant subtrees. The height of a subtree is, in turn, recursively defined as the branch length plus the average height of two descendant subtrees (the latter being zero for terminal nodes).

Using these models we calculated the Pearson correlation coefficients between the distance from the root to a given internal/external node and the value of the spacer length at a given node for all nodes in a tree. It should be noted that using just internal nodes in the correlation analysis does not change the results significantly. For the domain length analysis, the correlation was calculated between the evolutionary distance from the root and the sum of spacer length. The p-values for the correlation coefficients were estimated under the null hypothesis of being equal to zero; correlations for those families with p-values less than 0.01 were considered significant. The regression coefficients were calculated by linear regression analysis.

## Authors' contributions

YW and AP have made major contributions to conception, design analysis and interpretation of data. AP conceived of the study and wrote the first draft of the manuscript. TM was involved in the data analysis and revising the manuscript. VB participated in the statistical analysis and BS carried out comparisons with protein interactions. All authors have read and approved the final manuscript.
